# Supplementary material for: Multiple factors influence local perceptions of snow leopards and Himalayan wolves in the central Himalayas, Nepal
Source: PeerJ. 2020 Oct 15;8:e10108. doi: 10.7717/peerj.10108 (PMC7568854; doi:10.7717/peerj.10108)
Supplement: Supplemental Information 1 [file peerj-08-10108-s001.pdf]

Appendix S1. Questionnaire used for data collection.

Dear Sir/Madam,

I am a PhD student and studying snow leopards and wolves in Nepal. Many peoples, visitors in your area and, media/news are talking about problems with snow leopards and wolves in your area. As you are living close to carnivores in your area, I would like to know your opinion about carnivores in your region. The interview will not take more than 30 minutes (maximum). Please provide an answer sincerely as your information will help the concerned conservation authority to design future conservation activities in your region. I am not recording your name, therefore no one will be able to tally the answer given by you. There are no right or wrong answers, feel free to stop the interview anytime if you don't want to participate.

Thank you for your valuable time and for helping us with important information.

Note: The original questionnaire was in Nepali. The interview was conducted in Nepali or local languages (*Lobas* and *Gurung*) and later translated into English.

Form No.:…………. Date: …………

Respondent: Sex……………Age……………Literacy: Literate……………Illiterate……………...

Occupation: Agro-pastoralist……………… Livestock herding…………Others (specify)……..

Respondent village Name/Ward No. /Village Development Committee (VDC)……………………..

Surveyor Code: ………..... Date: …………….

---

1. How many livestock do you have?

Yak……… Jhopa……….Cow………. Mule………… Horse………… Goat………..Sheep……….. Remarks………………………

2. Have you lost any livestock from snow leopards and Wolves predation during July 2013 to June 2014? Yes…… No……, If yes, please specify total number………………………… (Total number)
The total number from Snow leopards predation: …………………
The total number from Wolves predation: …………………………

<u>Livestock loss Details:</u>
Livestock species: ………. Sex……….Age (in months)…….Killed/attack by………………………
Season: Which month………... Where: Corral/livestock pen…………………………………………
Pasture name…………………………………Name of the area……………………………………..

Habitat types: Cliff area……Riverbed……..Grassland….Scrubland……mixed scrubland…………
Others, specify……………………………………………………..

3. Did you report your losses to the Conservation Area Management Committee (CAMC) or Conservation authority? Yes ………… No ……………., If no, why……………………………......

4. Is there any incentive policy in your area against livestock losses due to carnivore predation?

5. Where do you graze your livestock? Can you please list down your grazing area?

Name of the pasture: ………………... Grazing months: (………………….to………………..months)

6. Have you seen snow leopards and wolves in your area? Yes…..No……, If Yes, where and when………………………………………………………………………………………………..

7. Do you like to see snow leopards and wolves in your areas? Do you think they are important to us?

Snow leopards: Yes……No……Don’t know……….
Wolves: Yes……..No……Don't know……………

8. If Yes, Why?
Snow leopards: ……………………………………………………………………………………….
Wolves: …………………………………………………………………………………………

9. If No, Why?
Snow leopards: ………………………………………………………………………………
Wolves: …………………………………………………………………………………………

10. Any other comments you would like to make with regards to conservation and conflict management?
………………………………………………………………………………………………..
………………………………………………………………………………………………..
………………………………………………………………………………………………..
