# Supplementary material for: Multiple factors influence local perceptions of snow leopards and Himalayan wolves in the central Himalayas, Nepal
Source: PeerJ. 2020 Oct 15;8:e10108. doi: 10.7717/peerj.10108 (PMC7568854; doi:10.7717/peerj.10108)
Supplement: Supplemental Information 2 [file peerj-08-10108-s002.pdf]

प्रिय सर/मैडम,

म विद्यावारिधी (पिएच.डी.) को विद्यार्थी हुँ र नेपालमा हिउँ चितुवा तथा ब्वाँसाहरूको अध्ययन गर्दैछु । तपाईंको क्षेत्रका धेरै व्यक्तिहरू, पर्यटक/आगन्तुकहरू र मिडिया/समाचारहरूले तपाईंको क्षेत्रमा हिउँ चितुवा र ब्वाँसाहरूसँगको समस्याको बारेमा कुरा गरिरहेका छन् । तपाईं आफ्नो क्षेत्रमा यी मांसाहारी वन्यजन्तुको नजिक बस्नुहुने भएकोले यी वन्यजन्तुको बारेमा तपाईंको राय बुझ्ने अपेक्षा मैले गरेको छु। यो अन्तर्वार्ता बढीमा ३० मिनेट भन्दा बढीको हुने छैन। कृपया आफूले थाहा पाएसम्म ईमानदारीपूर्वक जवाफ दिनुहोला किनकी तपाईंको महत्वपूर्ण ज्ञान तथा जानकारीले सम्बन्धित क्षेत्रको संरक्षण सम्बन्धी आधिकारिक निकायलाई तपाईंको क्षेत्रमा भविष्यमा संरक्षणका गतिविधिहरूको योजना तर्जुमा तथा कार्यान्वयन गर्न मद्दत मिल्नेछ । म तपाईंको नाम लेखीरहेको छैन, त्यसैले कसैले पनि तपाईंद्वारा दिइएको जवाफको स्रोत थाहा पाउन सक्ने छैन । यहाँ कुनै कुरामा पनि सही वा गलत उत्तरहरू भन्ने अर्थ लाग्ने व्यहोरा छैन । यदि तपाईंलाई अन्तर्वार्ताको दौरान यसलाई निरन्तरता दिन चाहनु हुन्न भन्ने लागेको खण्डमा कुनै पनि समयमा तपाईं अन्तर्वार्तालाई रोक्न सक्नुहुनेछ ।

तपाईंको बहुमूल्य समय र यस किसिमको महत्वपूर्ण जानकारी प्रदान गरिदिनु भई मद्दत गरिदिनु भएकोमा धन्यवाद दिन चाहन्छु ।

फारम नं. .... मितिः ....

उत्तरदाताः लिङ्ग .... उमेरः .... साक्षरताः साक्षर.............. निरक्षर.................

पेशाः कृषि-पशुपालन .... पशु चरन .... अन्य (उल्लेख गर्ने): ....

उत्तरदाताको गाउँको नाम/वडा नं./गाउँ विकास समिति (गा.वि.स.): ....

सर्वेक्षणकर्ताको संकेत/कोडः .... मितिः ....

---

१. तपाईंसँग कतिवटा पशुधन छन् ?

याक .... झोपा .... गाई ...... खच्चर .... घोडा .... च्यांग्रा .... भेडा .... कैफियत ....

२. जुलाई २०१३ देखि जुन २०१४ सम्ममा तपाईंले हिउँ चितुवा र ब्वाँसोको शिकारबाट कुनै पशुधन गुमाउनु भएको छ ? छ ..... छैन ....., यदि छ भने, कृपया कुल संख्या उल्लेख गर्नुहोस् .... (कुल संख्या)
हिउँ चितुवाको शिकारबाट गुमाउनु भएको पशुधनको संख्या ....
ब्वाँसोको शिकारबाट गुमाउनु भएको पशुधनको संख्या ....

पशुधनको क्षति विवरणः
पशुधनको प्रजाति वा प्रकार .... लिङ्ग .... उमेर (महिनामा): .... कुन वन्यजन्तुले मारेको/आक्रमण गरेको हो ....
मौसमः .... कुन महिना .... कहाँः ....कोरल/ पशुधन राख्ने खोर वा स्थल ....
चरन क्षेत्रको नामः .... सो क्षेत्रको नामः ....

वासस्थानको प्रकारः भिरालो क्षेत्र .... नदीतल क्षेत्र .... घाँसेभूमि.................. झाडीक्षेत्र .... मिश्रित झाडीक्षेत्र .... अन्य (उल्लेख गर्ने) ....

३. के तपाईंले आफ्ना क्षतिहरू बारे संरक्षण क्षेत्र व्यवस्थापन समिति वा संरक्षण सम्बन्धी आधिकारिक निकायलाई रिपोर्ट गर्नुभयो ? छ .... छैन .... यदि छैन भने किन ? ....

४. मांसाहारी वन्यजन्तुको कारणबाट हुने पशुधन क्षति विरूद्ध तपाईंको क्षेत्रमा कुनै प्रोत्साहन नीति छ ?

५. तपाईं आफ्नो पशुधन कहाँ चराउनुहुन्छ ? के तपाईं आफ्नो चरन क्षेत्रहरूको सूची तयार पार्न सक्नुहुन्छ ?
चरन क्षेत्रको नामः .... चराउने महिनाः (........................देखि ....)

६. के तपाईंले आफ्नो क्षेत्रमा हिउँ चितुवा र ब्वाँसो देख्नु भएको छ ? छ .... छैन..............
यदि छ भने, कहाँ र कहिले ....

७. के तपाईं आफ्नो क्षेत्रमा हिउँ चितुवा र ब्वाँसोहरू हेर्न मन पराउनुहुन्छ ? के तपाईंलाई ती हाम्रा लागि महत्वपूर्ण छन् जस्तो लाग्छ?
हिउँ चितुवाः छ .... छैन .... थाहा छैन ....
ब्वाँसोः छ .... छैन .... थाहा छैन ....

८. यदि छ भने, किन ? ....
हिउँ चितुवाः ....
ब्वाँसोः ....

९. यदि छैन भने, किन ? ....
हिउँ चितुवाः ....
ब्वाँसोः ....

१०. संरक्षण र द्वन्द्व व्यवस्थापन सम्बन्धमा कुनै अन्य टिप्पणीहरू तपाइँ गर्न चाहनुहुन्छ ?
....
....
....
