# Supplementary material for: Multiple factors influence local perceptions of snow leopards and Himalayan wolves in the central Himalayas, Nepal
Source: PeerJ. 2020 Oct 15;8:e10108. doi: 10.7717/peerj.10108 (PMC7568854; doi:10.7717/peerj.10108)
Supplement: Supplemental Information 5 [file peerj-08-10108-s005.docx]

**Supplemental File**

**Table S1: Model selection for perception towards the snow leopard.**

| **Model** | **df** | **logLik** | **AICc** | **delta** | **weight** |
| --- | --- | --- | --- | --- | --- |
| OWN + OCC + SEX + AGE * LIT | 9 | -233 | 485 | 0 | 0.45 |
| **OCC + SEX + AGE * LIT** | 8 | -234 | 485 | 0.27 | 0.39 |
| COMP * LOSS + OWN + OCC + SEX + AGE * LIT | 12 | -232 | 488 | 3.15 | 0.09 |
| COMP * LOSS + OCC + SEX + AGE * LIT | 11 | -234 | 490 | 5.02 | 0.04 |
| COMP * LOSS + OWN + OCC + SEX + AGE | 10 | -235 | 490 | 5.11 | 0.03 |
| OCC | 4 | -245 | 498 | 13.28 | 0 |
| COMP * LOSS + OWN + OCC | 8 | -242 | 499 | 14.47 | 0 |
| COMP * LOSS + OWN + OCC + SEX | 9 | -241 | 500 | 15.46 | 0 |
| AGE | 3 | -248 | 502 | 16.64 | 0 |
| LIT | 3 | -251 | 509 | 24.08 | 0 |
| COMP * LOSS + OWN + LIT | 7 | -248 | 511 | 26.11 | 0 |
| OWN | 3 | -255 | 516 | 31.06 | 0 |
| DIST | 3 | -255 | 516 | 31.12 | 0 |
| NULL | 2 | -257 | 518 | 32.70 | 0 |
| COMP * LOSS + OWN | 6 | -253 | 518 | 33.29 | 0 |
| COMP + LOSS * OWN | 6 | -253 | 519 | 33.74 | 0 |
| COMP + LOSS + OWN | 5 | -254 | 519 | 33.94 | 0 |
| SEX | 3 | -256 | 519 | 34.08 | 0 |
| COMP | 3 | -257 | 520 | 34.59 | 0 |
| LOSS | 3 | -257 | 520 | 34.65 | 0 |
| LOSS + COMP * OWN | 6 | -254 | 521 | 35.93 | 0 |
| COMP * LOSS | 5 | -256 | 522 | 36.65 | 0 |

**Notes.**

All continuous variables were standardized by 2 standard deviations (as per Gelman and Hill, 2007) and all models included a varying intercept on VDC (Village Development Committee). VDC is included as a random effect. AGE: age of the respondent, COMP: composition of the herd, i.e. proportion of large stock animals, DIST: distance from nearest conservation field office to respondent household, LIT: literacy (yes / no), LOSS: number of domestic animals lost to the snow leopard, OCC: respondent’s occupation (Herding, Agriculture and livestock farming, Others), OWN: number of domestic animals owned, SEX: gender of the respondent.

**Table S2 Model selection for perception towards the wolf.**

| **Model** | **df** | **logLik** | **AICc** | **delta** | **weight** |
| --- | --- | --- | --- | --- | --- |
| **COMP + DIST + LOSS + SEX + OWN** | 7 | -104 | 223 | 0 | 0.70 |
| COMP * LOSS + OWN + OCC + SEX | 9 | -105 | 228 | 5.22 | 0.05 |
| SEX | 3 | -111 | 229 | 5.62 | 0.04 |
| OWN | 3 | -112 | 229 | 6.22 | 0.03 |
| COMP + LOSS + OWN | 5 | -110 | 229 | 6.26 | 0.03 |
| COMP + LOSS + OWN + DIST | 6 | -109 | 230 | 6.64 | 0.03 |
| COMP * LOSS + OWN + OCC + SEX + AGE | 10 | -105 | 230 | 7.31 | 0.02 |
| OWN + OCC + SEX + AGE * LIT | 9 | -106 | 231 | 7.56 | 0.02 |
| COMP + LOSS * OWN | 6 | -109 | 231 | 7.67 | 0.02 |
| COMP * LOSS + OWN + LIT | 7 | -108 | 231 | 7.83 | 0.01 |
| COMP * LOSS + OWN | 6 | -109 | 231 | 8.1 | 0.01 |
| LOSS + COMP * OWN | 6 | -110 | 231 | 8.29 | 0.01 |
| NULL | 2 | -114 | 232 | 9.13 | 0.01 |
| LIT | 3 | -113 | 232 | 9.22 | 0.01 |
| DIST | 3 | -113 | 233 | 9.82 | 0.01 |
| COMP * LOSS + OWN + OCC + SEX + AGE * LIT | 12 | -104 | 233 | 9.91 | 0 |
| LOSS | 3 | -114 | 234 | 10.54 | 0 |
| AGE | 3 | -114 | 234 | 11.03 | 0 |
| COMP | 3 | -114 | 234 | 11.12 | 0 |
| OCC | 4 | -113 | 235 | 11.78 | 0 |
| OCC + SEX + AGE * LIT | 8 | -109 | 235 | 11.86 | 0 |
| COMP * LOSS + OWN + OCC | 8 | -109 | 235 | 11.96 | 0 |
| COMP * LOSS | 5 | -114 | 237 | 14.12 | 0 |
| COMP * LOSS + OCC + SEX + AGE * LIT | 11 | -109 | 240 | 17.22 | 0 |

**Notes.**

All continuous variables were standardized by 2 standard deviations (as per Gelman and Hill, 2007) and all models included a varying intercept on VDC (Village Development Committee). VDC is included as a random effect. AGE: age of the respondent, COMP: composition of the herd, i.e. proportion of large stock animals, DIST: distance from nearest conservation field office to respondent household, LIT: literacy (yes / no), LOSS: number of domestic animals lost to the Himalayan wolf, OCC: respondent’s occupation (Herding, Agriculture and livestock farming, Others), OWN: number of domestic animals owned, SEX: gender of the respondent.
